# Clinical and imaging characteristics of 4H syndrome: A case report

**DOI:** 10.1111/cns.13790

**Published:** 2021-12-24

**Authors:** Chao Wang, Shan Wang, Huanhuan Xie, Siyu Yang

**Affiliations:** ^1^ Department of Radiology The Second Affiliated Hospital, Zhejiang University School of Medicine NO.88 Jiefang Road Hangzhou China

## CONFLICT OF INTEREST

The authors have no conflicts to declare.

## CONSENT TO PARTICIPATE

The patient provided written consent for participation.

## CONSENT FOR PUBLICATION

The patient provided written consent for disclosure of medical information and images.

Dear Editors,

The term 4H syndrome was coined in 2006 and characterized by hypomyelination, hypodontia, and hypogonadotropic hypogonadism, which described a rare progressive hypomyelinating leukodystrophy.^1^ The 4H syndrome is now known to belong to a group of Pol III‐associated leukodystrophies caused by the mutations in the POLR3A or POLR3B gene.^2^, ^3^ Hypomyelination can be found in 95% of cases, but the extra‐neurological manifestations, such as hypodontia and hypogonadotropic hypogonadism, may be an absence that is not necessary for diagnosis.^2^, ^3^ There is wide variation in the presentation of 4H syndrome. In general, patients with POLR3A gene mutations are more severe than patients with POLR3B gene mutations.^2^ Herein, we report an uncommon case of 4H syndrome in a 26‐year‐old woman caused by novel heterozygous mutations in the POLR3A gene. This case deepens our understanding of the clinical and imaging characteristics of 4H syndrome.

This study followed the tenets of the Declaration of Helsinki and was performed according to the guidelines of the Second Affiliated Hospital of Zhejiang University School of Medicine. Written informed consent was obtained from the patient. A 26‐year‐old woman was admitted to the Department of Neurology, the Second Affiliated Hospital, Zhejiang University School of Medicine because of a 15‐year history of limbs and head tremors, unsteady walking, decreased intelligence. Her illness was so progressive that she had to drop out of high school. She had been inarticulate since two years ago and had urinary incontinence since last year. Recently, she had difficulty pronouncing words, and she could not walk by herself due to tremor progress. She was admitted to the neurology department for an assessment, where she was found to have not menstruated yet, and her secondary sexual characteristics were not developed. The detailed endocrine tests were further performed, including estradiol (68 pmol/L), luteinizing hormone (<0.3 IU/L), follicle‐stimulating hormone (<0.1 IU/L), progesterone (1.28 nmol/L), testosterone (1.16 nmol/L), and prolactin (145.4 mIU/L). Her bilateral mandibular second premolars were absent. Besides, her Heel‐Knee‐Tibia test and Romberg test were positive.

**FIGURE 1:**
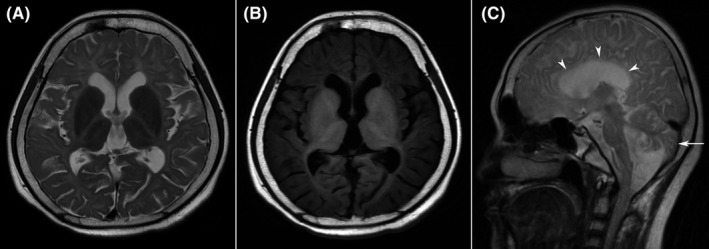
Extensive signal abnormalities of the whole white matter appearing hyperintense on T2 axial‐weighted image (A) and hypointense on T1 axial‐weighted image (B). The area of myelinated white matter is reduced. However, the volume of basal ganglia is normal (A, B). T2‐weighted midline sagittal image showed corpus callosum hypoplasia (arrowheads) and cerebellar atrophy (arrow) (C)

**FIGURE 2:**
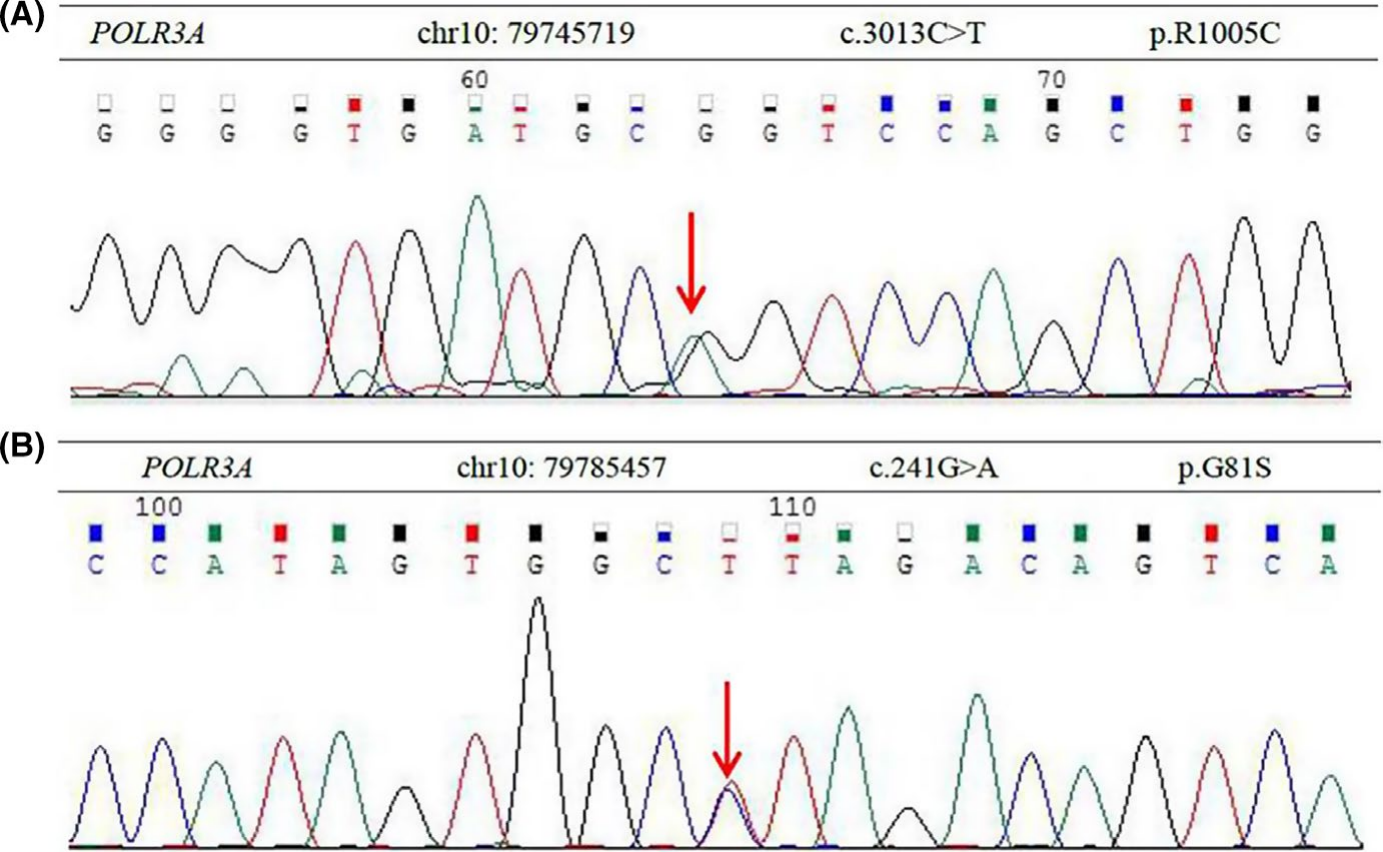
A heterozygous missense mutation in exon 23 of POLR3A gene (c.3013C>T, p.R1005C) contributing to amino acid substitution of cysteine for arginine at codon 1005 (A), and a heterozygous missense mutation in exon 3 of POLR3A gene (c.241G>A, p.G81S) contributing to amino acid substitution of serine for glycine at codon 81 were detected (B)

Brain MRI revealed extensive signal abnormalities of the whole white matter appearing hyperintense on T2‐weighted image (T2WI) and hypointense on T1WI consistent with hypomyelination. Hypomyelination lesions involved the deep white matter, internal and external capsule, and corpus callosum. In addition, cerebellar atrophy and hypoplastic corpus callosum were detected (Figure 1). Combined the primary amenorrhea, hypodontia, with the hypomyelination on brain MRI, this patient was considered as a suspected case of the 4H syndrome. Then, targeted sequencing analysis was further performed for the definitive diagnosis. A heterozygous missense mutation in exon 23 of POLR3A gene (c.3013C>T, p.R1005C) contributing to amino acid substitution of cysteine for arginine at codon 1005 and a heterozygous missense mutation in exon 3 of POLR3A gene (c.241G>A, p.G81S) contributing to amino acid substitution of serine for glycine at codon 81 were detected (Figure 2). According to the clinical manifestations, brain MRI characteristics, and result of targeted sequencing analysis, the final diagnosis of the 4H syndrome was made due to the mutations in the POLR3A gene. After admission, symptomatic treatment was given for this patient, including improving tremor symptoms and mood. Her symptoms were slightly better than before, and she was discharged 12 days later. And she was recommended to be on regular follow‐up.

Congenital hypomyelinating disease is a rare heterogeneous central nervous system leukoencephalopathy, in which Pol III‐associated leukoencephalopathies manifest as different combinations of motor dysfunction, abnormal dentition, and hypogonadotropic hypogonadism. In recent years, these syndromes have been thought to be caused by mutations involving the POLR3A, POLR3B, and POLR1C genes and are inherited as autosomal recessive disorders.^4^ Besides the 4H syndrome, the Pol III‐associated leukoencephalopathies include four other syndromes, including (1) the leukodystrophy with oligodontia (LO); (2) tremor‐ataxia with central hypomyelination (TACH); (3) ataxia, delayed dentition, and hypomyelination (ADDH); and (4) hypomyelination with cerebellar atrophy and hypoplasia of the corpus callosum (HCAHC). Apart from hypodontia and primary amenorrhea, our case showed tremor‐ataxia, cerebellar atrophy, and corpus callosum hypoplasia.

MRI is currently the best diagnostic imaging method for the evaluation of hypomyelinated leukoencephalopathy. Typically, brain MRI shows extensive signal abnormalities of the whole white matter, including the centrum semiovale, corona radiate, internal, and external capsules.^2^, ^5^, ^6^ The bilateral cerebral white matter typically demonstrates hyperintense on T2WI and hypointense on T1WI, indicating diffuse hypomyelination. Histopathological examination reveals a myelin deficiency, a decrease in the number of oligodendrocytes, and a loss of neurons proportional to the degree of myelin involvement. The loss of oligodendroglia is most severe in the white matter of the centrum semiovale, while the preservation of oligodendroglia is greatest in the white matter around capillaries, arterioles, and venules. In addition, the loss of axons was moderate in the centrum semiovale, and the axons close to blood vessels were well preserved.^2^, ^7^ Hypomyelination combined with cerebellar atrophy, T2 hypointensity in the ventrolateral thalamus, and myelination of the pyramidal tract in the posterior limb of the internal capsule, optic radiation, and dentate nucleus are present in most cases.^8^, ^9^ In our case, brain MRI showed diffuse cerebral hypomyelination with the reduced area of myelinated white matter, manifesting as the atrophy of cerebral white matter. However, the volume of basal ganglia is normal. The corpus callosum is typically normal in children, but the corpus callosum is always thinned in adults, reflecting a slowly progressive reduction in the volume of white matter.^2^, ^8^, ^9^ In addition, a previous study showed cerebellar atrophy was regarded as an essential imaging manifestation.^8^ In the present case, both cerebellar atrophy and corpus callosum hypoplasia were shown.

In this study, we report a case presenting with the 4H syndrome due to novel heterozygous missense mutations in the exon of the POLR3A gene. MRI showed extensive cerebral hypomyelination with cerebellar atrophy and corpus callosum hypoplasia. There is still no effective treatment for this disorder after getting a definitive diagnosis. However, the definitive diagnosis can avoid the need for keeping going to the doctors and provide a predictable prognosis for the patient's family, thereby facilitating appropriate long‐term care planning. Therefore, it is of great importance for radiologists and neurologists to recognize the clinical and imaging characteristics of this disorder, make the correct diagnosis in time, and avoid delayed treatment. Targeted sequencing analysis should be offered to the suspected patients in time.

## Data Availability

The data that support the findings of this study are available from the corresponding author upon reasonable request.
